# Hypoglycemic and Hypolipidemic Effects of *Phellinus Linteus* Mycelial Extract from Solid-State Culture in A Rat Model of Type 2 Diabetes

**DOI:** 10.3390/nu11020296

**Published:** 2019-01-30

**Authors:** Yangyang Liu, Chaorui Wang, Jinshan Li, Yuxia Mei, Yunxiang Liang

**Affiliations:** 1 State Key Laboratory of Agricultural Microbiology, College of Life Science and Technology, Huazhong Agricultural University, Wuhan 430070, China; m13026100673@163.com (Y.L.); wang_rui@webmail.hzau.edu.cn (C.W.); kingsun@mail.hzau.edu.cn (J.L.); mei@mail.hzau.edu.cn (Y.M.); 2 Department of Chemistry, University of California, Davis, CA 95616, USA

**Keywords:** *Phellinus linteus* extract, type 2 diabetes, hypoglycemic effect, hypolipidemic effect

## Abstract

Hypoglycemic and hypolipidemic effects of *P. linteus* have been observed in numerous studies, but the underlying molecular mechanisms are unclear. In this study, we prepared *P. linteus* extract (PLE) from mycelia of solid-state culture, and evaluated its hypoglycemic and hypolipidemic effects in rat models of high-fat diet (HFD)-induced and low-dose streptozotocin (STZ)-induced type 2 diabetes. PLE treatment effectively reduced blood glucose levels, and improved insulin resistance and lipid and lipoprotein profiles. The hypoglycemic effect of PLE was based on inhibition of key hepatic gluconeogenesis enzymes (FBPase, G6Pase) expression and hepatic glycogen degradation, and consequent reduction of hepatic glucose production. PLE also: (i) enhanced expression of CPT1A and ACOX1 (key proteins involved in fatty acid β-oxidation) and low-density lipoprotein receptor (LDLR) in liver, thus promoting clearance of triglycerides and LDL-C; (ii) inhibited expression of 3-hydroxy-3-methylglutaryl-coenzyme A reductase (HMGCR) in liver, thus reducing cholesterol production; (iii) displayed strong hepatoprotective and renal protective effects. Our findings indicate that PLE has strong potential functional food application in adjuvant treatment of type 2 diabetes with dyslipidemia.

## 1. Introduction

Diabetes is a metabolic disease characterized by high blood glucose level (hyperglycemia) resulting from insulin secretion deficiency, insulin resistance, or these two factors in combination [1]. It causes progressive harmful effects to kidneys, heart and other organs, with associated diseases (cardiovascular, renal, and others), and reduces quality of life for patients [1,2]. Blood glucose and lipid homeostasis are maintained by various metabolic processes occurring in the liver, including glycogenesis, glycogen synthesis/degradation, glycolysis, and fatty acid β-oxidation [3,4]. Hyperglycemia can be controlled by inhibition of liver gluconeogenesis and glycogen degradation, and by promotion of glycogen synthesis and glucose glycolysis [5,6,7]. Enhancement of β-oxidation process in liver helps normalize blood lipid profile and hepatic insulin sensitivity [8,9].

Diabetes treatment strategies in common use typically have undesirable side effects and complications that are difficult to control [10,11]. Increasing research attention has been paid to novel, effective anti-diabetes agents derived from natural sources [5,12]. One major source is medicinal mushroom species, which are easily cultivated and rich in beneficial active ingredients. Numerous studies have focused on *Ganoderma lucidum*, *Inonotus obliquus*, and *Phellinus linteus*, whose active ingredients have been shown to effectively inhibit obesity induced by high-fat diet in rodent models [13,14], and to reduce hyperglycemia [15,16,17]. *P. linteus* [18] has a >2000-year history of applications in traditional Chinese medicine, and is widely used in many eastern Asian countries as a functional food [19]. It is rich in polysaccharides and other small molecules, and has well documented anti-cancer [20], anti-oxidative [21,22], anti-inflammatory [23,24,25], hepatoprotective [26], and antibacterial [27] effects. The active component of *P. linteus* lowers blood glucose level. Kim, D.’s group demonstrated that exopolysaccharides extracted from *P. linteus* grown by liquid fermentation had hypoglycemic and hypolipidemic effects and ameliorated liver damage [28]. Kim, H.’s group found that mycelial polysaccharides of *P. linteus* under submerged fermentation inhibited expression of inflammatory cytokines IFN-γ and TNF-α, and development of diabetes in non-obese diabetic (NOD) mice [29]. Polysaccharides from mycelia and hot water extracts of fruiting bodies of *P. linteus* were useful for diabetes treatment by reducing oxidative damage of islet cells, promoting insulin secretion, and enhancing insulin resistance [17,30,31]. The small molecule hispidin and phenolic compounds purified from *P. linteus* were also useful for diabetes treatment [32,33,34].

Under natural conditions, *P. linteus* has a 2- to 3-year growth cycle, which is too slow to meet medicinal demand [18]. Studies to date focused on treatment of diabetes mellitus have used *P. linteus* under liquid fermentation as the source of experimental extracts, but *P. linteus* under solid-state culture has not been utilized for this purpose. Numerous studies have described the hypoglycemic effect of *P. linteus*; however, these studies were performed in vitro or in some animal model of type 1 diabetes [28,29,30]. In the present study, hypoglycemic and hypolipidemic effects, and underlying molecular mechanisms, of mycelial *P. linteus* extract (PLE) from solid-state culture were evaluated in type 2 diabetic rat models based on high-fat diet or low-dose streptozotocin treatment.

## 2. Materials and Methods

### 2.1. Materials and Reagents

*P. linteus* was obtained from the Microbial Genetic Stock Center of Huazhong Agricultural University (Wuhan, China). *P. linteus* mycelia from solid-state culture were lyophilized and ground to powder. Streptozotocin (STZ) was from Sigma-Aldrich (St. Louis, MO, USA). Metformin hydrochloride was from Yuekang Pharmaceutical Group Co. (Beijing, China). Other reagents were of analytical grade and from Sinopharm Chemical Reagent Co. (Shanghai, China).

### 2.2. Preparation of P. linteus Extract (PLE)

Dried *P. linteus* mycelia powder as above was extracted for 3 h in hot water (90 °C) in proportion 1:40 (*w*/*v*), twice [20]. Insoluble particles in water extract were separated by tube centrifugation (10,000× *g*) to obtain supernatant, which was then concentrated and mixed with 4-fold volume of pure ethanol at 4 °C overnight. Precipitate was redissolved in water, concentrated, and lyophilized to obtain PLE (i.e., polysaccharide-enriched powder).

### 2.3. Component Analysis of PLE

Total polysaccharide content of PLE was determined by phenol-sulfuric acid method, with dextran as standard [35]. Total protein content was determined by Lowry method, with BSA as standard [36]. Total flavone content was determined as described previously [37].

### 2.4. Animals

Male Sprague-Dawley rats (150–180 g) were from the Experimental Animal Center of Disease Prevention and Control in Hubei province (Wuhan, China). All experimental animal procedures were approved by the Animal Care and Use Committee of Huazhong Agricultural University (protocol # SCXK2011-0012), and performed in accordance with internationally accepted guidelines and ethical principles. Animals were maintained on 12 h/12 h dark/light cycle at 22 °C with *ad lib* access to standard lab food pellets and water, and left to adapt to this environment for 1 week prior to experiments.

### 2.5. Induction of Type 2 Diabetes and Experimental Design

7 rats were randomly selected as normal control (NC) group with “Chow” diet, and 43 rats were fed high-fat diet (HFD) (cat # D12451, Opensource Animal Diets; Changzhou, China) (Supplementary Table S1). After 6 weeks, the HFD group, in comparison with NC group, had 50 g higher mean body weight, higher fasting serum triglyceride and total cholesterol levels, and impaired glucose tolerance (Supplementary Table S2). Diabetes was induced in HFD animals by i.p. injection of low-dose STZ (45 mg/kg·bw) after 8 h fasting [38,39]. 1 week later, fasting blood glucose (FBG) level was measured by AccuChek active glucometer (Roche, Switzerland), and animals with FBG levels >11.1 mmol/L were selected for further grouping.

Based on the results of the pre-experiment (Supplementary Figure S1), this study was performed at the doses of 300 and 600 mg/kg·bw. Diabetic rats were divided randomly into four groups (each with *n* = 7): model control group (termed “Model”) (0.9% saline); metformin-treated group (“Met”) (metformin (a drug commonly used for treatment of type 2 diabetes), 120 mg/kg·bw); low-dose PLE group (“L”) (PLE, 300 mg/kg·bw); high-dose PLE group (“H”) (PLE, 600 mg/kg·bw). Polysaccharide content in PLE was used as standard for intragastric dose, and animals received this dose by intragastric gavage once per day, at a fixed time, for 8 weeks. FBG level was measured every 2 weeks following the 8 weeks of intragastric administration.

### 2.6. Collection and Preservation of Experimental Samples

Following the final administration, animals were fasted for 8 h, then anesthetized using pentobarbital sodium. Whole blood was taken from retinal vein by glass capillary, and serum was separated by centrifugation (5 min, 4 °C, 3000 r·min^−1^) and stored at −20 °C. Liver was removed rapidly, rinsed with normal saline, and immersed in liquid nitrogen (−80 °C) for long-term storage.

### 2.7. Serum Biochemical Indexes

Serum biochemical indexes were determined using the following assay kits from Jiancheng Bioengineering Institute (Nanjing, China), as per the respective manufacturer’s protocols: total bilirubin (Bil) (# C019); alkaline phosphatase (ALP) (# A059); alanine aminotransferase (ALT) (# C009); aspartate aminotransferase (AST) (# C010); liver glycogen (# A043); uric acid (UA) (# C012); urea nitrogen (BUN) (# C013); creatinine (Cre) (# C011); glycosylated serum protein (GSP) (# A037); triglyceride (TG) (# A110); total cholesterol (T-CHO) (# A111); low-density lipoprotein cholesterol (LDL-C) (# A113); free fatty acids (FFA) (# A042); high-density lipoprotein cholesterol (HDL-C) (# A112). Serum insulin was measured using insulin ELISA kit (Bio-Swamp Life Science; Shanghai). Homeostatic model assessment of insulin resistance (HOMA-IR) was performed as described previously [40].

### 2.8. Hepatic TG and T-CHO

Hepatic TG and T-CHO extracts were prepared by homogenizing 0.2 g of liver tissues in chloroform: methanol (2:1, *v*/*v*) [41]. Hepatic TG and T-CHO contents were measured by assay kits (# A110 and # A111).

### 2.9. RNA Extraction from Liver, and Quantitative Real-Time Polymerase Chain Reaction (Qrt-PCR)

Total RNA was extracted from liver using TRNzol Universal Reagent (cat # DP424, Tiangen Biotech (Beijing) Co., Beijing, China), and RNA quality was evaluated using Ultra-Micro UV spectrophotometer. mRNA was reverse-transcribed into cDNA using HiScript II Q RT SuperMix for qPCR kit (Vazyme Biotech Co., Nanjing, China). qRT-PCR amplification was performed using AceQ SYBR Green Master Mix kit (Vazyme) with three repeats for each reaction, as follows: 95 °C for 1 min, followed by 40 cycles of 95 °C for 10 s and 60 °C for 30 s. Detailed information is given in Supplementary Table S3 for qRT-PCR primers of fructose-1,6-bisphosphatase (FBPase), glucose-6-phosphatase (G6Pase), solute carrier family 2 member 2 (GLUT2), glucokinase (GCK), acyl-CoA oxidase 1 (ACOX1), carnitine palmitoyltransferase 1A (CPT1A), 3-hydroxy-3-methylglutaryl-CoA reductase (HMGCR), low-density lipoprotein receptor (LDLR), and β-actin. Relative gene expression was assessed by 2^−ΔΔCt^ method using β-actin as reference.

### 2.10. Statistical Analysis

Experimental data were expressed as mean ± SEM. Differences between means for various groups were analyzed by one-way Analysis of Variance (ANOVA) using the SPSS 19.0 software program (SPSS Inc.; Chicago, IL, USA) with least-square deconvolution (LSD). Differences with *p* < 0.05 and *p* < 0.01 were considered significant and highly significant, respectively.

## 3. Results

### 3.1. Main Components of PLE

PLE was obtained by hot-water extraction after grinding solid-state cultured *P. linteus* mycelia into powder. The major components of the PLE (comprising >90% of total biomass) were polysaccharides and proteins (Table 1). Flavonoids were a minor component (<4%).

### 3.2. PLE and Metformin Treatments Inhibited Overeating, Body Weight Loss, and FBG Level in Diabetic Rat Model

**Figure 1:**
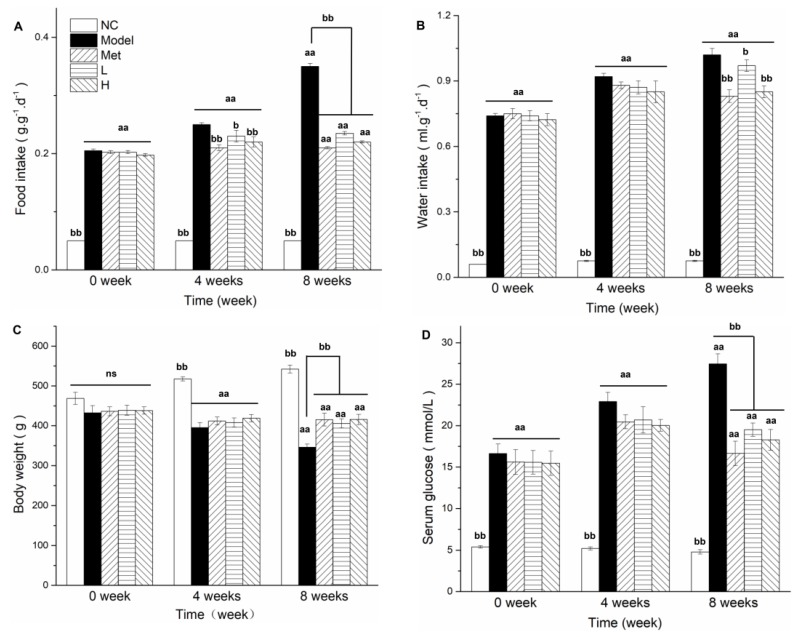
PLE and Metformin treatments inhibited overeating, body weight loss, and FBG level in diabetic rat model. Food intake (**A**), water intake (**B**), body weight (**C**), and FBG level (**D**) were measured at 0, 4, and 8 weeks. Data are expressed as mean ± SEM (*n* = 7 for each group). ^aa^ *p* < 0.01, ^a^ *p* < 0.05 for Model, Met, L, and H vs. NC. ^bb^ *p* < 0.01, ^b^ *p* < 0.05 for NC, Met, L, and H vs. Model. ns: no significance.

At week 0, the four experimental groups (Model, Met, L, H; see Section 2.5) did not differ significantly in food and water intake (Figure 1A,B), body weight (Figure 1C), or FBG level (Figure 1D). Food intake increased progressively in Model group until the end of the study period (i.e., week 8). Food intake in Met and PLE (i.e., L and H) groups remained stable from week 4 to week 8, and was significantly (*p* < 0.01) lower than that in Model group at week 8. Water intake had a trend similar to that of food intake. At week 4, body weight in all four experimental groups was lower than at week 0, and was significantly (*p* < 0.01) lower than in control (NC) group. Body weight did not differ significantly among the experimental groups. As of week 8, Model group showed the largest body weight loss, and body weights of Met and H groups were significantly (*p* < 0.01) higher than that of Model group. Diabetes-induced weight loss was inhibited by PLE or Met treatment. At week 4, FBG levels were much higher in the experimental groups than in NC group, but did not differ significantly among experimental groups (Figure 1B). At week 8, diabetes continued to worsen in Model group, but its progress was effectively delayed in Met and PLE groups. After administration for 8 weeks, the FBG levels of Met, L, and H groups were stable and significantly (*p* < 0.01) lower than that of Model group. These findings indicate that PLE treatment reduced FBG levels and ameliorated diabetes symptoms.

### 3.3. PLE and Metformin Treatments Reduced Glycosylated Serum Protein (GSP) Level, and Improved Insulin Resistance and Promoted Hepatic Glycogen Storage

**Figure 2:**
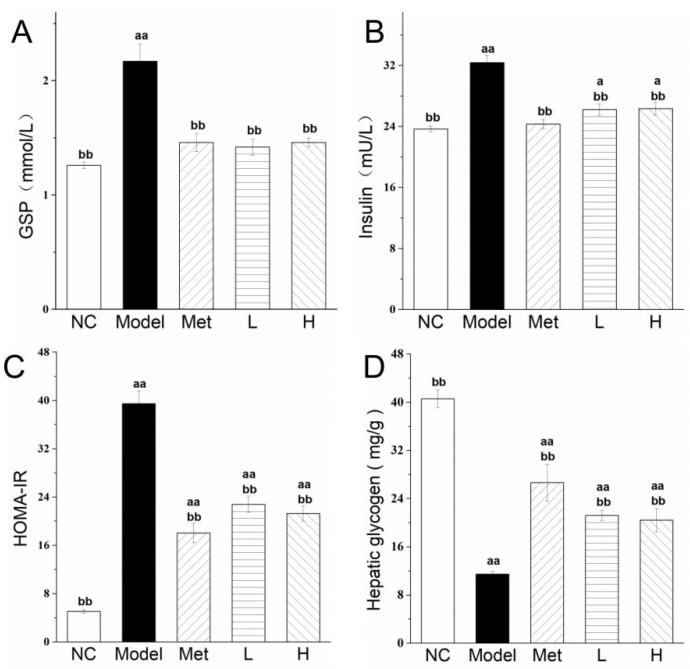
PLE and metformin treatments reduced glycosylated serum protein (GSP) level, and improved insulin resistance and promoted hepatic glycogen storage. Serum GSP level (**A**), insulin (**B**), HOMA-IR (**C**), and hepatic glycogen content (**D**). Statistical procedures and notations as in Figure 1.

Hypoglycemic effects of Met and PLE were evaluated by measuring GSP and serum insulin levels in the experimental groups. Changes in GSP level reflect fluctuations of blood glucose level. GSP level at week 8 was significantly (*p* < 0.05) higher for Model group than for NC group (Figure 2A). Average GSP levels in Met and PLE groups were higher than in NC group, but the difference was not significant (*p* > 0.05). GSP levels in Met, L, and H groups were significantly (*p* < 0.05) lower than in Model group, indicating a preferable hypoglycemic effect of PLE.

Insulin is the only hormone capable of reducing blood glucose level, and insulin resistance is a commonly observed phenomenon in type 2 diabetes patients. Fasting serum insulin level (Figure 2B) and HOMA-IR index (Figure 2C) were both significantly (*p* < 0.01) higher for Model group than for NC group, consistently with type 2 diabetes symptoms. At week 8, fasting serum insulin level and HOMA-IR index were significantly (*p* < 0.01) lower in Met and PLE groups than in Model group. These findings indicate that both PLE and Met effectively ameliorated insulin resistance in our type 2 diabetes rat model. Liver glycogen content in Model group was ~28% (*p* < 0.01) of that in NC group (Figure 2D). Liver glycogen contents of Met, L, and H groups were significantly (*p* < 0.05) higher than that of Model group, because of improving insulin resistance. Thus, PLE treatment effectively dampened blood glucose fluctuation by reducing GSP level and improving insulin resistance. The target of PLE in regulation of blood glucose level is presumably related to inhibition of hepatic glucose output.

### 3.4. PLE and Metformin Treatments Improved Serum Lipid and Lipoprotein Profiles

**Figure 3:**
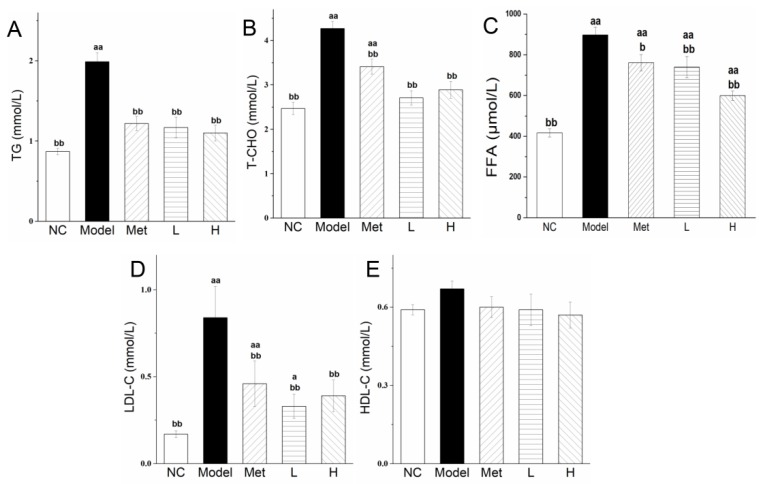
PLE and metformin treatments improved serum lipid and lipoprotein profiles. Serum TG (**A**), T-CHO (**B**), FFA (**C**), LDL-C (**D**)and HDL-C (**E**) levels. Statistical procedures and notations as in Figure 1.

Abnormal lipid metabolism was observed in our diabetic rat model under conditions of abnormal glucose metabolism. Serum TG, T-CHO, FFA and LDL-C levels were significantly (*p* < 0.01) higher in Model group than in NC group (Figure 3A–D). TG, T-CHO and FFA levels were significantly (*p* < 0.01) lower in L and H groups than in Model group. LDL-C levels were significantly (*p* < 0.01) lower in Met and PLE groups than in Model group. PLE was more effective than Met in improving lipid profiles. HDL levels did not differ significantly among the various groups (Figure 3E). Based on the data of serum TG, T-CHO, FFA, LDL-C, we could conclude that the regulation of lipid metabolism in the diabetic rats had been disordered. PLE and metformin treatments effectively improved serum lipid and lipoprotein profiles

### 3.5. PLE and Metformin Regulated Expression of Key Gluconeogenesis and Glycolysis Enzymes in Liver

**Figure 4:**
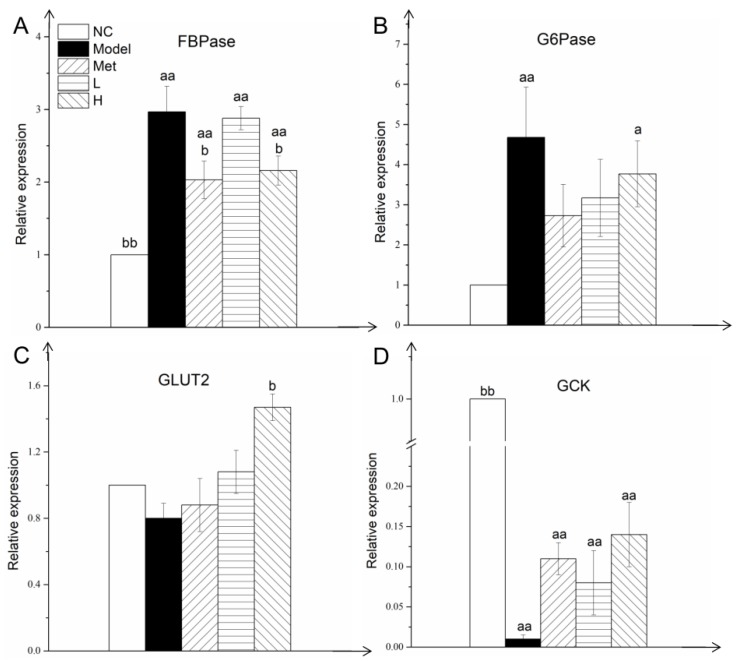
PLE and metformin regulated expression of key gluconeogenesis and glycolysis enzymes in liver. FBPase (**A**), G6Pase (**B**), GLUT2 (**C**), and GCK (**D**) expression. Statistical procedures and notations as in Figure 1.

The molecular mechanism underlying the hypoglycemic effect of PLE was examined by measuring expression levels of key enzymes involved in gluconeogenesis and glycolysis. Upregulated expression of FBPase and G6Pase, the key gluconeogenesis enzymes in liver, leads to increased hepatic glucose production and consequent hyperglycemia. Expression levels of liver FBPase (Figure 4A) and G6Pase (Figure 4B) were significantly (*p* < 0.01) higher in Model group than in NC group; consequently, Model group showed higher liver gluconeogenesis and transfer of glucose into bloodstream. Met is a clinical anti-diabetes drug that reduces blood glucose level by improving insulin resistance and inhibiting hepatic glucose production. At week 8, FBPase and G6Pase expression levels were lower in Met and PLE groups than in Model group, and the difference was significant (*p* < 0.05) in the case of FBPase in H and Met groups.

PLE treatment also affected glucose transporter 2 (GLUT2) expression. GLUT2 expression was significantly (*p* < 0.05) higher in H group than in Model group (Figure 4C), resulting in enhanced transfer of glucose from blood to liver and its conversion into glycogen for storage. Expression of GCK, a rate-limiting enzyme of glycolysis, was significantly (*p* < 0.01) lower in Model group than in NC group (Figure 4D). GCK expression was higher in Met and PLE groups than in Model group, but the difference was not significant. These findings, taken together, suggest that the hypoglycemic effect of PLE is due primarily to inhibited expression of key gluconeogenesis enzymes in liver, with consequent reduction of hepatic glucose output and enhancement of glucose utilization and clearance.

### 3.6. PLE and Metformin Decreased Hepatic TG and T-CHO Contents and Regulated Expression of Key Enzymes of Lipid Metabolism in Liver

**Figure 5:**
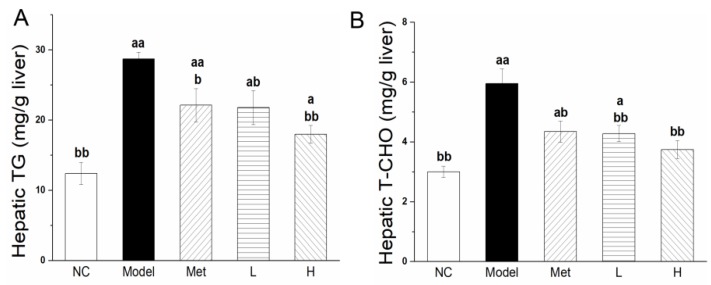
PLE and metformin decreased hepatic TG and T-CHO content. Hepatic TG (**A**) and hepatic T-CHO (**B**) contents. Statistical procedures and notations as in Figure 1.

**Figure 6:**
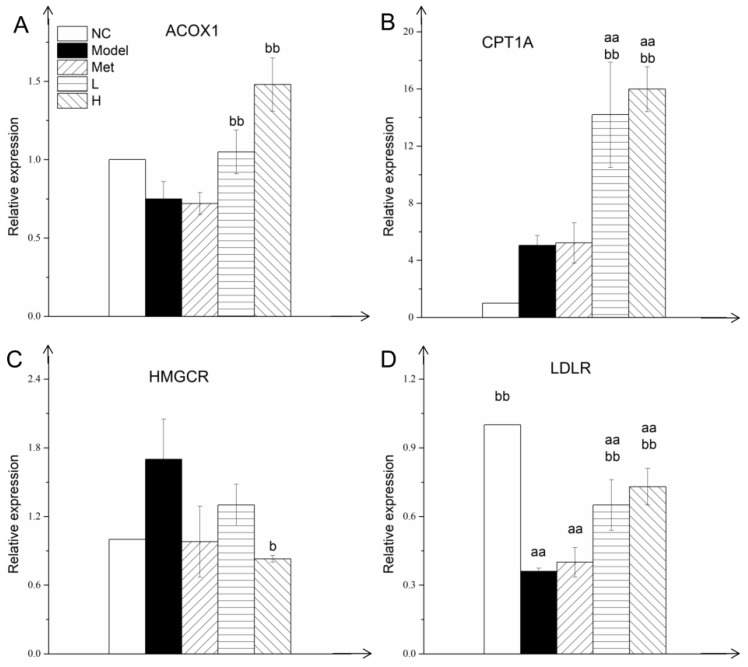
PLE and metformin regulated expression of key enzymes of lipid metabolism in liver. ACOX1 (**A**), CPT1A (**B**), HMGCR (**C**), and LDLR (**D**) expression. Statistical procedures and notations as in Figure 1.

Treatment with Met or PLE reduced both hyperglycemia and accompanying hyperlipidemia in our diabetic rat model. After intervention with metformin and PLE for 8 weeks, the hepatic TG (Figure 5A) and T-CHO (Figure 5B) levels in the Model group were significantly higher than the NC group. Compared with the Model group, the intervention of metformin and PLE could reduce the TG and T-CHO contents in the liver, but did not reach the NC group level. Promotion of β-oxidation processes in liver reduces lipid accumulation and enhances hepatic insulin sensitivity. ACOX1 and CPT1A are key proteins involved in the fatty acid β-oxidation process. Expression of ACOX1 (Figure 6A) and CPT1A (Figure 6B) in L and H groups was significantly (*p* < 0.01) higher than in Model group. Increased expression of these proteins in liver reduces fat storage. Cholesterol is synthesized and metabolized primarily in liver, and HMGCR is the key enzyme in its synthesis process. HMGCR expression level in Model group was 1.6-fold higher than in NC group (Figure 6C), resulting in higher cholesterol synthesis. HMGCR expression in H group was significantly (*p* < 0.05) lower than in Model group. Thus, PLE reduced cholesterol synthesis and blood cholesterol level by inhibiting HMGCR expression. LDLR expression in liver tissue helps clear cholesterol from the blood. LDLR expression was significantly (*p* < 0.01) lower in Model group than in NC group (Figure 6D). LDLR expression was significantly (*p* < 0.01) higher in both L and H groups than in Model group. These findings, taken together, indicate that PLE treatment improves blood lipid and lipoprotein profiles by regulating expression of lipid metabolic enzymes in liver.

### 3.7. PLE and Metformin Ameliorate Liver Injury and Kidney Injury

Liver injury typically results in elevated levels of hepatic markers (AST, ALT, ALP, bilirubin). Diabetic rats (Model group), in comparison with NC group, showed significantly (*p* < 0.05) higher levels of these four markers (Table 2), and severe liver injury. Met and PLE treatment ameliorated liver injury relative to Model group. ALT level in L and H groups was restored to values similar to those in NC group (*p* > 0.05). The hepatoprotective effect of PLE was stronger than that of Met. Diabetic nephropathy is a major complication of diabetes. Levels of related renal function indicators in serum were examined. Levels of blood uric acid, Cre, and BUN were significantly (*p* < 0.05) higher in Model group than in NC group (Table 2). Degree of severity of kidney injury was in the order Model group > Met group > PLE groups. These findings indicate that PLE had a substantial liver protective effect, and ameliorated diabetes-related kidney injury.

## 4. Discussion

**Figure 7:**
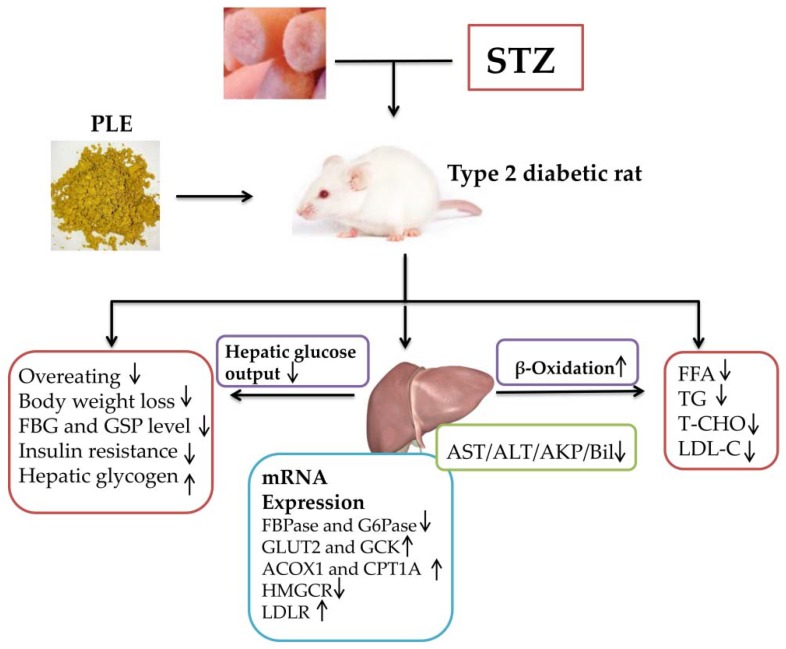
Schematic model of PLE hypoglycemic and hypolipidemic effects. PLE intervention effectively alleviated liver damage and insulin resistance caused by high-fat diet and STZ. Concomitantly, PLE reduced FBG by inhibiting liver glucose output and improved lipid profile by promoting liver fatty acid oxidation.

Numerous reports have described hypoglycemic effects of *P. linteus* [17,18,29]; however, most such studies were performed in vitro or in animal models of type 1 diabetes. However, type 2 diabetes is far more common than type 1. Furthermore, in most studies of *P. linteus* effects on diabetes mellitus, the experimental materials used were mycelia from liquid fermentation, or fruiting bodies from wild mushrooms. However, in view of the prolonged growth cycle of *P. linteus*, such sources are not adequate for medicinal demand [20]. Solid-state culture techniques, in comparison with submerged fermentation, give higher yields and/or superior product characteristics [42]. Mycelia from solid-state culture are a good source material. We evaluated hypoglycemic and hypolipidemic effects of PLE from solid-state culture mycelia using rat models of HFD-induced and low-dose-STZ-induced type 2 diabetes. This study shows that PLE can effectively alleviate liver damage caused by high-fat diet and STZ, improve insulin resistance, inhibit hepatic glucose output, promote liver fatty acid oxidation, and achieve regulation of blood glucose and blood lipids (Figure 7).

High-fat diet and STZ-induced rats were characterized by increased fasting blood glucose, increased food intake, and increased water intake [5,43] and weight loss [38,44]. Deficiency of insulin or decreased sensitivity of muscle tissue to insulin leads to degradation of muscle tissue protein and weight loss [44,45,46]. Fat mobilization of adipose tissue caused by deficiency of insulin or insulin resistance also results in weight loss and increasing blood lipids [45,47]. After the intervention of metformin and PLE for 4weeks, the body weight of the rats remained stable, which was significantly better than that of the Model group (Figure 1C) at the 8th week. This may be related to the improvement of insulin resistance (Figure 2C). The decreased feeding (Figure 1A) and drinking (Figure 1B) in diabetic rats both benefitted from the hypoglycemic effect of PLE.

The liver plays many crucial roles in the maintenance of blood glucose and lipid homeostasis [48]. Blood glucose homeostasis depends on appropriate balance of glycolysis, gluconeogenesis, and glycogen metabolism [49]. FBPase, the rate-limiting enzyme in gluconeogenesis, catalyzes dephosphorylation of fructose 1,6-bisphosphate to fructose 6-phosphate [49]. The terminal step in hepatic gluconeogenesis and glycogenolysis, production of free glucose, is catalyzed by glucose-6-phosphatase [50]. These two enzymes are involved in the process of hepatic glycogen production in the liver, and their expression levels are regulated by insulin signaling [51]. In diabetic individual, the activity and expression levels of these two enzymes in the liver are up-regulated [49]. *Ganoderma lucidum* extract has been found to reduce blood glucose level by inhibiting expression of liver gluconeogenesis enzymes and reducing glucose production [15,38,52,53]. PLE similarly inhibits FBPase and G6Pase expression in liver, and reduces gluconeogenesis and glucose production. PLE also enhances expression of key glycolytic enzyme glucokinase (GK) and of GLUT2, with consequent improvement of glucose utilization and clearance [54].

HFD can induces liver fat deposition, and excessive fat deposition can cause insulin resistance [39,55]. The abnormal hepatic insulin signaling is associated with the dysregulation mitochondrial fatty acid oxidation [56]. The β-oxidation process is the major fatty acid catabolism pathway in liver and the acceleration of fatty acid oxidation process will help to clear excess hepatic lipids and restore its insulin sensitivity [57,58]. ACOX1 and CPT1A are the rate-limiting enzymes of β-oxidation in peroxisomes and mitochondria, respectively [8], and HMGCR is the rate-limiting cholesterol synthesis enzyme in liver [53]. They play an important role in liver lipid metabolism. In STZ-induced diabetes, hepatic LDLR mRNA expression is strongly reduced, in association with increased serum cholesterol level [59]. Enhanced expression of ACOX1 [38], CPT1A [60], and LDLR [61], and reduced HMGCR expression [53], jointly maintain lipid homeostasis. Dai’s group found that baicalin (a flavonoid from the herbal medicine *Scutellaria baicalensis*) can directly activate hepatic CPT1 to accelerate the lipid influx into mitochondria for oxidation and ameliorate diet-induced hepatic steatosis and insulin resistance [62]. Flavonoid-rich Chinese bayberry (*Morella rubra Sieb. et Zucc.*) fruit extract can directly regulate gene expression of liver lipid synthase and reduce liver lipid content in diabetic mice [63]. PLE also contains flavonoids (Table 1, 3.87%), which may be the substance that directly regulates the expression of liver lipid metabolism enzymes. The molecular mechanisms whereby PLE reduced blood lipid level (Figure 3) and hepatic lipid level (Figure 5), shown in the present study, to be correlated with upregulation of ACOX1 (Figure 6A), CPT1A (Figure 6B), and LDLR expression (Figure 6C) and inhibition of HMGCR expression (Figure 6D). The exact function requires the purification of the PLE extract for further research. The hypoglycemic effect of PLE also improves hepatic lipid clearance [28].

STZ-induced diabetes is often accompanied by abnormal liver function, which is known to aggravate naturally occurring diabetes [64,65]. Persistent or chronic hyperglycemia results in severe microvascular complications and consequent kidney injury [66]. Serum biochemical indexes of liver and kidney injury were effectively reduced by PLE treatment, presumably because of its anti-oxidative [26,67] and anti-inflammatory effects [68].

Polysaccharide [17,29] and small molecular components [69] extracted from *P. linteus* have long been used for prevention or treatment of diabetes. The primary active components of *P. linteus* involved in regulation of blood glucose level remain to be clearly identified.

Mushrooms are rich in polysaccharides, many of which are non-digestible [70]. Certain microbes in the mammalian gut have the potential to break down complex polysaccharides to release energy [71]. Development of sequencing technology during the past decade has facilitated detailed studies of gut microbes. Occurrence and development of obesity and diabetes are clearly related to changes in composition of gut microbes [72]. Many reports have documented the ability of fungal polysaccharides to slow or reverse metabolic syndrome through their effects on gut microbes. Polysaccharides from *G. lucidum* and *Hirsutella sinensis* reduced HFD-induced obesity and improved insulin resistance in mice [13,73]. They also helped slow or reverse metabolic syndrome by correcting intestinal microbial disorders associated with HFD, promoting integrity of intestinal epithelia, and reducing chronic inflammation. *P. linteus* polysaccharides ameliorated HFD-induced and high-fructose-diet-induced insulin resistance in mice by regulating intestinal flora involved in vitamin B12 synthesis [31]. Studies are underway to elucidate the detailed mechanisms whereby *P. linteus* polysaccharides help control diabetes through their regulatory effects on intestinal microbes.

In conclusion, the findings presented here indicate that PLE reduces blood glucose level and enhances insulin resistance and glycogen storage in a diabetic rat model. The blood glucose reducing effect was based on inhibition of mRNA expression for key hepatic enzymes involved in glycogen degradation and glycogenesis. PLE also helped maintain homeostasis of lipid and lipoprotein profiles by promoting expression of key β-oxidation enzymes and LDLR in liver, inhibited HMGCR expression, and ameliorated diabetes-associated liver and kidney injury.

## Figures and Tables

**Table 1:** Major components of *Phellinus linteus* extract (PLE).

Component	Content (%)	Detection Method
Polysaccharides	62.56%	Phenol-sulfuric acid method
Proteins	28.34%	Lowry method
Flavonoids	3.87%	Aluminum chloride spectrophotometric method

**Table 2:** PLE and metformin ameliorate liver injury and kidney injury.

Parameter	Group				
	NC	Model	Met	L	H
AST (IU/L)	14.99 ± 1.27 ^bb^	46.27 ± 6.39 ^aa^	23.55 ± 2.31 ^a,bb^	19.76 ± 1.68 ^bb^	17.13±1.87 ^bb^
ALT (IU/L)	27.51 ± 4.58 ^b^	123.17 ± 16.61 ^a^	43.86 ± 4.40 ^b^	43.32 ± 6.53 ^b^	36.55 ± 2.68 ^b^
AKP (U/L)	27.93 ± 1.99 ^bb^	102.12 ± 6.37 ^aa^	64.24 ± 6.76 ^aa,bb^	58.18 ± 6.10 ^a,bb^	62.34 ± 6.08 ^aa,bb^
Bil (μmol/L)	1.80 ± 0.19 ^b^	6.13 ± 0.89 ^a^	5.91 ± 1.06	5.41 ± 0.89	4.96 ± 0.93
UA (mol/L)	111.43 ± 1.52 ^b^	164.08 ± 8.97 ^a^	134.63 ± 10.67	116.60 ± 7.69 ^b^	110.66 ± 5.80 ^bb^
Cre (μmol/L)	45.79 ± 3.55 ^bb^	71.46 ± 2.86 ^aa^	60.06 ± 5.76	53.61 ± 3.18 ^b^	49.46 ± 2.59 ^bb^
BUN (mmol/L)	2.21 ± 0.15 ^bb^	7.15 ± 0.58 ^aa^	6.48 ± 0.46 ^aa^	6.08 ± 0.30 ^aa^	4.86 ± 0.12 ^aa^

Data are expressed as mean ± SEM (*n* = 7 for each group). ^aa^ *p* < 0.01, ^a^ *p* < 0.05 for Model, Met, L, and H vs. NC. ^bb^ *p* < 0.01, ^b^ *p* < 0.05 for NC, Met, L, and H vs. Model.

## Supplementary Materials

The following are available online at http://www.mdpi.com/2072-6643/11/2/296/s1, Table S1: Composition of HFD (cat # D12451, Opensource Animal Diets), Table S2: Values of body weight, fasting blood glucose (FBG), triglycerides (TG), and total cholesterol (T-CHO) at 6 wk in animals fed on standard Chow diet and HFD, Table S3: Primer sequences for qRT-PCR, Figure S1: PLE and metformin treatments reduced FBP(A), GSP(B), TG(C) and T-CHO(D) level. Data are expressed as mean ± SEM (*n* = 7 for each group). ^aa^ *p* < 0.01, ^a^ *p* < 0.05 for Model, Met, L, and H vs. NC. ^bb^ *p* < 0.01, ^b^ *p* < 0.05 for NC, Met, L, and H vs. Model.
