# Supplementary material for: Hypoglycemic and Hypolipidemic Effects of Phellinus Linteus Mycelial Extract from Solid-State Culture in A Rat Model of Type 2 Diabetes
Source: Nutrients. 2019 Jan 30;11(2):296. doi: 10.3390/nu11020296 (PMC6412584; doi:10.3390/nu11020296)
Supplement: Supplementary file 1 [file nutrients-11-00296-s001.pdf]

Supplemental Table S1. Composition of HFD (cat # D12451, Opensource Animal Diets).

Component	Composition	
	gm%	kcal%
Protein	24	20
Carbohydrate	41	35
Fat	24	45
Total		100
kcal/gm	4.73	
Ingredient	gm	kcal
Casein	200	800
L-Cystine	3	12
Corn starch	72.8	291
Maltodextrin	100	400
Sucrose	172.8	691
Cellulose	50	0
Soybean oil	25	225
Lard	177.5	1598
Mineral mix S10026	10	0
Dicalcium Phosphate	13	0
Calcium carbonate	5.5	0
Potassium citrate, 1 H2O	16.5	0
Vitamin mix V10001	10	40
Choline bitartrate	2	0
FD&C Yellow dye #5		
FD&C Red dye #40	0.05	0
FD&C Blue dye #1		
Total	858.15	4057

Supplemental Table S2. Values of body weight, fasting blood glucose (FBG), triglycerides (TG), and total cholesterol (T-CHO) at 6 wk in animals fed on standard Chow diet and HFD.

**Parameter**	**Group**	
	**Chow diet**	**HFD**
Body weight (g)	448.44±13.41	504.83±5.86[aa]
FBG (mmol/L)	5.79±0.15	7.24±0.09[aa]
TG (mmol/L)	0.70±0.04	1.14±0.05[aa]
T-CHO (mmol/L)	1.42±0.07	2.43±0.09[aa]

At wk 6, animals were fasted for eight h, weighed, and FBG, TG, and T-CHO values were measured from a blood sample from tail tip. Each of the four parameters was significantly higher in HFD group than in Chow diet (NC) group. Differences between means were analyzed by unpaired two-tailed Student's t-test. [aa] $p < 0.01$.

Supplemental Table S3. Primer sequences for qRT-PCR.

Target	Reference sequence number	Primers (5'—3')	Product length
β-actin	NM_031144.3	F: TCCTAGCACCATGAAGATCAAG<br>R: GACTCATCGTACTCCTGCTTG	132 bp
Liver fructose-1,6-bisphosphatase (FBPase)	NM_012558.3	F: CTATGCTACCTGTGTCCTTGTG<br>R: AGGCAGTCGATGTTGGATG	123 bp
Glucose-6-phosphatase, catalytic subunit (G6Pase)	NM_013098.2	F: AATGAACGTGCTCCACGACT<br>R: CTGCCACCCAGAGGAGATTG	185 bp
Glucokinase (GCK)	NM_012565.2	F: CTTCACCTTCTCCTTCCCTG<br>R: ATCTCAAAGTCCCCTCTCCT	150 bp
Solute carrier family 2 member 2 (GLUT2)	NM_012879.2	F: CACATCCTACTTGGCCTATCTG<br>R: TCAGTGCCCCTTAGTCTTTTC	146 bp
Acyl-CoA oxidase 1 (ACOX1)	NM_017340.2	F: CTGCCACATATGACCCCAAGA<br>R: GCATGTAACCCGTAGCACTCT	154 bp
Carnitine palmitoyltransferase 1A (CPT1A)	NM_031559.2	F: GATGGCAGAGGCTCACCAAG<br>R: TGATGCCATTCTTGAACCGGA	152 bp
3-hydroxy-3-methylglutaryl-CoA reductase (HMGCR)	NM_013134.2	F: CTTGGTGGCCTCCATTGAGAT<br>R: AGTGTCACCGTTCCCACAAT	130 bp
Low-density lipoprotein receptor (LDLR)	NM_175762.2	F: ACATTTCGGGTCTGTGATCC<br>R: CGTTCTTGCCACATGAATCTTC	139 bp

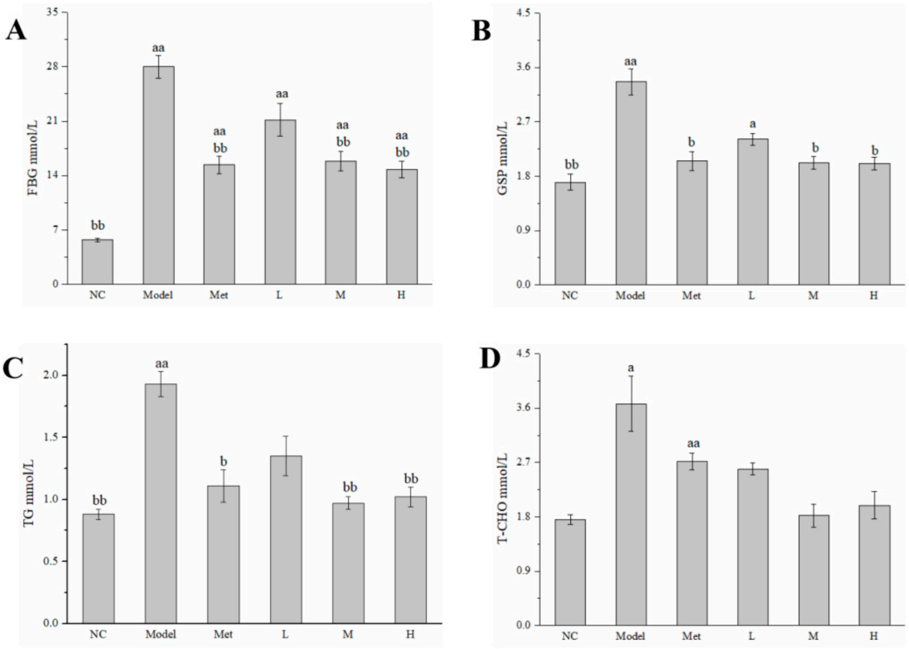

Sfigure.1 PLE and metformin treatments reduced FBP(A), GSP(B), TG(C) and T-CHO(D) level

Data are expressed as mean ± SEM (n=7 for each group). ^{aa} p< 0.01, ^{a} p< 0.05 for Model, Met, L, and H vs. NC. ^{bb} p< 0.01, ^{b} p< 0.05 for NC, Met, L, and H vs. Model.
